# A Dialysis Membrane-Integrated Microfluidic Device for Controlled Drug Retention and Nutrient Supply

**DOI:** 10.3390/mi16070745

**Published:** 2025-06-25

**Authors:** Hajime Miyashita, Yuya Ito, Kenta Shinha, Hiroko Nakamura, Hiroshi Kimura

**Affiliations:** 1 Department of Mechanical Engineering, School of Engineering, Tokai University, 4-1-1 Kitakaname, Hiratsuka 259-1292, Japan; 2 Micro/Nano Technology Center (MNTC), Tokai University, 4-1-1 Kitakaname, Hiratsuka 259-1292, Japan

**Keywords:** microphysiological systems, microfluidic device, dialysis membrane, co-culture, drug assay

## Abstract

Traditional pre-clinical drug evaluation methods, including animal experiments and static cell cultures using human-derived cells, face critical limitations such as interspecies differences, ethical concerns, and poor physiological relevance. More recently, microphysiological systems (MPSs) that use microfluidic devices to mimic in vivo conditions have emerged as promising platforms. By enabling perfusion cell culture and incorporating human-derived cells, MPSs can evaluate drug efficacy and toxicity in a more human-relevant manner. However, standard MPS protocols rely on discrete medium changes, causing abrupt changes in drug concentrations that do not reflect the continuous pharmacokinetics seen in vivo. To overcome this limitation, we developed a Dialysis Membrane-integrated Microfluidic Device (DMiMD) which maintains continuous drug concentrations through selective medium change via a dialysis membrane. The membrane’s molecular weight cut-off (MWCO) enables the retention of high-molecular-weight drugs while facilitating the passage of essential low-molecular-weight nutrients such as glucose. We validated the membrane’s molecular selectivity and confirmed effective nutrient supply using cells. Additionally, anticancer drug efficacy was evaluated under continuously changing drug concentrations, demonstrating that the DMiMD successfully mimics in vivo drug exposure dynamics. These results indicate that the DMiMD offers a robust in vitro platform for accurate assessment of drug efficacy and toxicity, bridging the gap between conventional static assays and the physiological complexities of the human body.

## 1. Introduction

Pre-clinical studies in the drug discovery process are conducted using animal or cell-culture tests with human-derived cells to investigate a new drug candidate’s efficacy, toxicity, and pharmacokinetics. However, within the field of animal testing, issues arise related to species differences between experimental animals and humans, in addition to ethical concerns [1]. Cell-culture tests involving the use of human-derived cells are constrained by difficulties in reproducing the in vivo environment and confirming organ interactions; in addition, the extrapolation of results to humans is limited, making it challenging to evaluate drug efficacy accurately [2]. There is thus an urgent need to develop a novel drug efficacy evaluation system that can be used in pre-clinical studies to replace conventional animal tests and cell culture tests using human-derived cells.

A novel cell culture platform referred to as the “Microphysiological System (MPS)” based on microfluidics has recently attracted considerable research attention [3,4]. The MPS is an in vitro model that mimics the human in vivo environment. It is equipped with medium perfusion functions, with microfluidic devices and a chamber capable of culturing human-derived cells [5]. The dynamic environment provided by the perfusion in the MPS can maintain the function and morphology of cultured cells and reproduce physiological functions with greater efficacy than conventional cell culture tests involving the use of human-derived cells [6]. Moreover, the human-derived cells used in the MPS enable the evaluation of human-specific drug responses that cannot be predicted through animal testing. The MPS is designed to mimic the functions of specific organs or tissues, and can evaluate their specific functions in vitro. In addition, complex interactions between different organs can be evaluated by co-culturing cells from multiple organs in the MPS [7]. Based on the above findings, the MPS may be expected to serve as a powerful tool for evaluating new drugs’ efficacy, toxicity, and pharmacokinetics. However, in drug efficacy evaluations using the MPS, the medium is typically changed every 1 or 2 days to supply nutrients and remove waste products to facilitate cell maintenance. Drug concentrations in the MPS change significantly because the anticancer drug in the medium is replaced simultaneously with the medium. In the in vivo environment, as the drug is absorbed, distributed, metabolized, and excreted, its concentration changes continuously over time. Reproducing continuous changes in drug concentration is important for accurately predicting a drug’s efficacy, toxicity, and pharmacokinetics because the efficacy and duration of a drug depend on changes in drug concentration.

To date, several approaches have been proposed to improve accuracy in predicting drug efficacy and toxicity in vitro by mimicking in vivo drug concentration changes using the MPS. Petreus et al. developed a platform to accurately assess the drug efficacy of tumor spheroids by sequentially delivering eight concentrations of the drug at a constant flow rate [8]. In addition, Singh et al. demonstrated that the automation of drug injection systems allows for the simulation of long-term drug concentration changes in mice [9]. Furthermore, Guerrero et al. utilized computer control to replicate in vivo drug concentration changes in mice and humans, and obtained results which demonstrated the impact of drug exposure time on efficacy [10]. All of these systems face the common challenges of difficulty in retaining drugs from one-way flow and inability to assess the impact of changes in metabolite concentrations.

In the present study, we focused on integrating a dialysis membrane into the MPS to address these issues. The molecular selectivity of the dialysis membrane determines the size of molecules that pass through based on the Molecular Weight Cut-Off (MWCO), allowing for the retention of substances by molecular weight [11]. Imura et al. demonstrated that integrating dialysis membranes into the MPS enables retention of high-molecular-weight drugs and effective waste removal [12]. This finding demonstrates that drug retention by the dialysis membrane mimics continuous in vivo drug concentration changes, making it useful for evaluating drug activity. However, the developed MPS is a single-tumor cell culture system, making it difficult to evaluate drug efficacy and toxicity based on metabolites through co-culture with metabolically active cells. Furthermore, drug efficacy evaluation through the co-culture of multiple cell types requires sufficient nutrient supply for cell maintenance, with a novel system being required to retain drugs and evaluate their efficacy.

In light of the above, we developed a Dialysis Membrane-integrated Microfluidic Device (DMiMD) that maintains continuous drug concentrations by use of the medium change method through dialysis membranes, with the aim of establishing an in vitro system that mimics continuous in vivo drug concentration changes. In this study, the evaluations of the DMiMD included assessing its molecular selectivity through dialysis membranes, evaluating nutrient supply performance, and testing the efficacy of anticancer drugs using HepG2 or Upcyte liver model cells and A549 human lung cancer cells targeted by anticancer drugs. The results of the molecular selectivity evaluation confirmed that the dialysis membrane separates and retains high-molecular-weight substances while selectively supplying low-molecular-weight substances, such as glucose, which are essential for cell culture. The results of evaluating nutrient supply performance using HepG2 and A549 cells showed that the medium change method through the dialysis membranes supports cell culture similarly to the conventional method. The evaluation of anticancer drug efficacy showed that medium change through dialysis membranes allows for drug efficacy assessment under continuously changing drug concentration conditions, demonstrating that the DMiMD is an effective tool for evaluating drug efficacy and toxicity.

## 2. Materials and Methods

### 2.1. Dialysis Membrane-Integrated Microfluidic Device (DMiMD)

**Figure 1:**
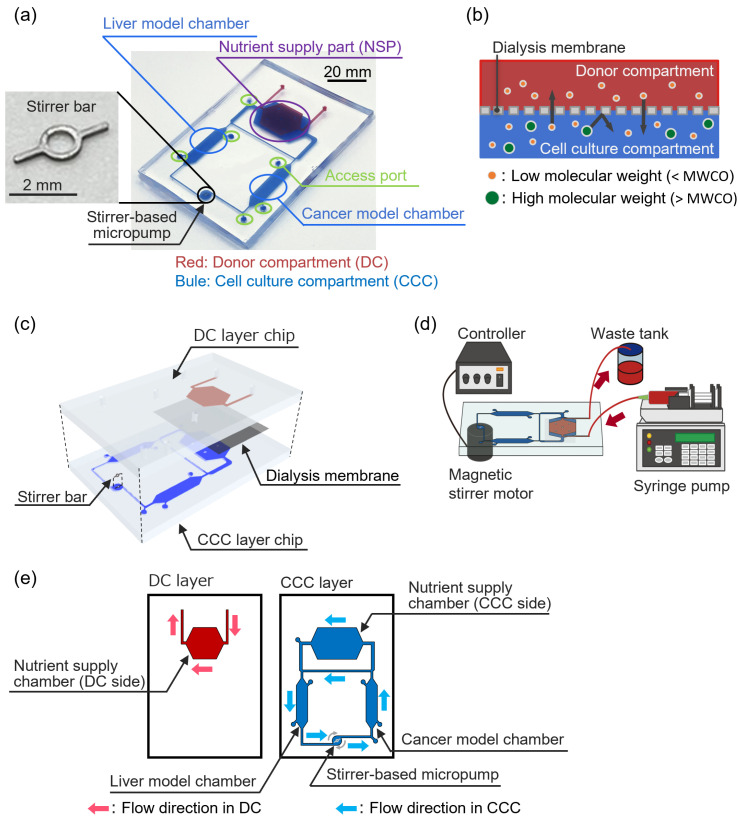
A Dialysis Membrane-integrated Microfluidic Device (DMiMD). (**a**) Schematic illustration of the DMiMD. The DMiMD consists of a cell culture compartment (CCC) (microfluidic channels, stirrer-based micropump, and organ model chambers) and a donor compartment (DC). (**b**) Cross-sectional view of the nutrient supply part (NSP) in the DMiMD. The NSP is separated into the CCC and the DC by a dialysis membrane, allowing substances to be retained based on their molecular weight. (**c**) Schematic of an enlarged view of the DMiMD, which was assembled by bonding the DC layer and CCC layer chips with a dialysis membrane sandwiched between them. (**d**) Experimental setup of the DMiMD. To supply nutrients from the DC to the CCC through the dialysis membrane, the medium in the DC was introduced using a syringe pump. A stirrer motor was installed beneath the DMiMD to drive the stirrer-based micropump which perfused the medium in the CCC. (**e**) Flow direction of the DMiMD. Fresh culture medium was delivered to the DC by a syringe pump, and a stirrer pump perfused the culture medium in the CCC. The arrows indicate the flow direction in each compartment and the rotation direction of the stirrer bar.

Our DMiMD was based on a cell culture microfluidic device developed by Kimura et al. [13] This consisted of a cell culture compartment (CCC) and a donor compartment (DC) (Figure 1a). The CCC consisted of two organ model chambers, a nutrient supply part (NSP), and a stirrer-based pump connected by a microfluidic channel. The volume of the CCC was roughly 166.5 µL. The NSP was separated into chambers of the CCC and the DC by a dialysis membrane (area: 200 mm^2^) (Figure 1b). The molecular weight of glucose, which is an essential nutrient for cell culture, is 180.16 [14]. The DMiMD was therefore integrated with a dialysis membrane (F35-7935, Cellulose Tubes for Osmotic Experiments, Narika, Tokyo, Japan) with an MWCO of 3.5 × 10^3^ that efficiently permeates glucose. The dialysis membrane was characterized by a pore size of 0.0024 μm and an MWCO of 3.5 × 10^3^, facilitating efficient glucose permeation.

Nutrients such as glucose in a perfused culture medium in the DC diffuse to the CCC based on a difference in concentration via the dialysis membrane at the NSP. While drugs and proteins with molecular weights greater than the MWCO are retained in the CCC by the dialysis membrane, even when the medium flows through the DC. The DMiMD can therefore retain drugs in the CCC while supplying nutrients to the cultured cells. In addition, the stirrer-based micropump developed in our previous study can generate flow in the microfluidic channel by rotating the built-in stirrer bar in response to the rotation of a magnetic stirrer motor installed beneath the DMiMD [15]. In the previously developed device, a configuration was adopted in which the culture medium flows from the intestinal model chamber to the liver model chamber, mimicking the physiological pathway in humans whereby nutrients such as glucose, absorbed in the intestine, reach the liver via the portal vein [13]. Similarly, the DMiMD was designed in such a way that nutrients from the DC are first supplied to the liver model chamber and subsequently reach the cancer model chamber.

The CCC possessed access ports with silicone tubes for cell maintenance. As detailed further below, cells were seeded into the CCC through access ports using syringes, and the same ports were used for washing and for medium change.

The DMiMD was fabricated by bonding the CCC layer and DC layer chips made of polydimethylpolysiloxane (PDMS, DOWSIL™SILPOT 184 W/C, Dow Toray, Tokyo, Japan) (Figure 1c). The DC layer and CCC layer chips were molded using a mold with microchannels patterned via photolithography [16,17]. The microchannel height of the DC and CCC layers was 0.3 mm. The DC layer chip and the CCC layer chip were bonded across the dialysis membrane. The dialysis membrane was silanized to ensure strong chemical bonding with the PDMS [18]. The DC layer and CCC layer chips were oxygen plasma-treated at the bonding surface using a plasma cleaner (PDC-32C, Harick plasma, Ithaca, NY, USA) and then bonded. A stainless-steel stirrer bar (3 mm long; 0.2 mm wide) was used for the stirrer-based micropump in the DMiMD. A different version of the device was also fabricated with a tissue culture-treated dish plate (I3020-100, AGC TECHNO GLASS, Shizuoka, Japan) that was processed using a laser machine (zing24, Laser Connect, Tokyo, Japan) on the bottom of the organ model chambers for culturing cells with weak adhesion. The DMiMD and the processed plate were bonded with double-sided tape (760H #25, Teraoka Seisakusho, Tokyo, Japan). The DMiMD was single-use, and was disposed of after each experiment.

### 2.2. Cell Culture

HepG2 (JCRB1054, JCRB Cell Bank, Osaka, Japan) and Upcyte (KHE001-10-03, Upcyte Technologies, Hamburg, Germany) were used as liver model cells, and A549 (RCB0098, RIKEN Bio Resource Research Center, Ibaraki, Japan) cells were used as human lung cancer cells. These cells were cultured in an incubator at 37 °C with 5% CO_2_. HepG2 and A549 cells were cultured in DMEM low glucose (10320-032, Dulbecco’s Modified Eagle Medium, Thermo Fisher Scientific-JP, Tokyo, Japan) supplemented with 10% fetal bovine serum (FBS, Bio West, Tokyo, Japan), 1% non-essential amino acids (NEAA, 11140–050, Thermo Fisher Scientific-JP, Tokyo, Japan), and 1% Antibiotic–Antimycotic solution (161–23181, FUJIFILM Wako, Osaka, Japan). Upcyte cells were cultured in Upcyte Hepatocyte High-Performance Medium (MHE003, Upcyte Technologies, Hamburg, Germany) supplemented with 10% FBS, 1% NEAA, and 1% Antibiotic–Antimycotic solution.

Before cell seeding, the CCC was coated with collagen I-P (170323, Nitta Gelatin, Osaka, Japan). HepG2 or Upcyte cells were seeded at a 1.0 × 10^5^ cells/cm^2^ density into the liver model chamber as the liver model via the access ports. HepG2 cells were pretreated with 50 µM Rifampicin (13292-46-1, Tokyo Chemical Industry, Tokyo, Japan) for 3 days to induce CYP3A4 during the evaluation of anticancer drug efficacy [19,20]. After 3 days of confluence in liver model cells, A549 cells were seeded at a 5.0 × 10^4^ cells/cm^2^ density into the cancer model chamber as the cancer model.

The medium in the CCC was unchanged, and nutrients were supplied from the DC via the dialysis membrane at the NSP. The medium in the DC was pumped at a flow rate of 1.0 µL/min using a syringe pump (EUDC24B8, Minato Concept, Tokyo, Japan) (Figure 1d). The stirrer-based micropump was driven by the magnetic stirrer motor installed beneath the DMiMD, with a 3.0 µL/min flow rate. The flow direction in each compartment is shown in Figure 1e.

### 2.3. Cell Density Evaluation

Cell viability was evaluated using a cell staining method. Cells in the DMiMD were stained with Hoechst 33342 (346-07951, Dojindo Laboratories, Kumamoto, Japan) to determine total nuclei, and PI (341-07881, Dojindo Laboratories, Kumamoto, Japan) to determine dead nuclei. The total number of cells and the number of dead cells were determined from fluorescence images obtained using a fluorescent microscope. The cell density of live cells was calculated from the difference between these values.

### 2.4. Quantification of Fluorescent Substances

The amount of fluorescent substance in the CCC was quantified from fluorescence images obtained using a research stereomicroscope (SMZ-25, Nikon Corporation, Tokyo, Japan). First, solutions with varying fluorescent concentrations were placed in the CCC, and calibration curves of fluorescent concentrations and brightness values were generated from fluorescence observation. Thereafter, the brightness values of the fluorescence images of the CCC were measured, and the concentration of fluorescent substance was calculated based on the obtained brightness values using the calibration curve.

### 2.5. Statistical Analysis

All values were expressed as the mean ± SD of at least three independent experiments. Statistical analysis was performed using GraphPad Prism (version 10.3.0). Data were evaluated using the Tukey–Kramer test, and statistical significance was determined at *p* < 0.05.

## 3. Results and Discussion

### 3.1. Evaluation of Molecular Selectivity Using the DMiMD

To investigate the molecular selectivity of the dialysis membrane in the DMiMD, we evaluated the changes in substance concentrations in the CCC when substances of different molecular weights were introduced into the DC. When fresh medium was introduced into the DC using a syringe pump in the DMiMD, substances with a molecular weight lower than that of the MWCO of the dialysis membrane diffused into the CCC through the membrane. In addition, substances with a molecular weight greater than the MWCO of the dialysis membrane were retained and did not permeate through it. The DMiMD was integrated with the dialysis membrane with an MWCO of 3.5 × 10^3^, enabling sufficient permeation of glucose (molecular weight: 180.16), an essential nutrient for cell culture. In this experiment, medium containing 50 µM Uranine (molecular weight: 376) (F0096, Tokyo Chemical Industry, Tokyo, Japan) and 50 µM Dextran (molecular weight: 2.0 × 10^4^) (FD20S, Sigma-Aldrich Japan, Tokyo, Japan) was introduced to the DC using the syringe pump.

**Figure 2:**
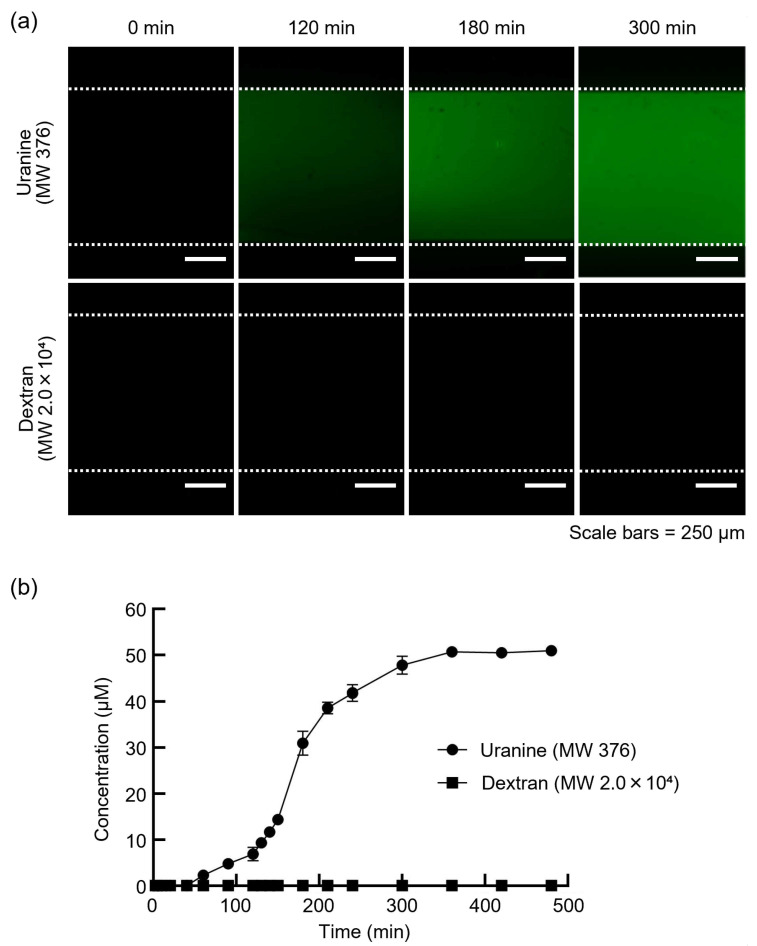
Evaluation of molecular selectivity in the DMiMD. (**a**) Comparisons of the amounts of substance permeated by the difference in molecular weight in the DMiMD. (**b**) Concentrations of Uranine and Dextran fluorophores in the CCC over time were calculated from the luminance values of the fluorescence images. The concentration of Uranine in the CCC increased over time; in comparison, the concentration of Dextran remained unchanged.

In the CCC, the fluorescence of Uranine increased over time. In contrast, the fluorescence of Dextran was barely visible (Figure 2a). As a result of calculating the changes in fluorescence substance concentrations, the concentration of Uranine in the CCC increased over time. The concentration of Dextran in the CCC remained almost zero until the end of the experiment (Figure 2b). After 360 min, the concentration of Uranine in the CCC reached 50 µM, identical to that of the fluorescent substance solution introduced to the DC.

The molecular weight of Uranine was less than that of the MWCO of the dialysis membrane; in comparison, the molecular weight of Dextran was greater. The temporal concentration changes of Uranine and Dextran in the CCC indicated that Uranine permeated and diffused from the DC to the CCC, whereas Dextran remained in the DC without permeating the membrane. These findings demonstrated that the DMiMD with molecular selectivity enables the permeation and retention of molecules. Glucose is an essential nutrient for cell culture. The molecular weight of glucose is lower than that of Uranine. Glucose therefore permeates the dialysis membrane in the DMiMD, enabling continuous nutrient supply to the CCC through the membrane by pumping fresh medium to the DC.

### 3.2. Evaluation of Nutrient Supply Performance Using the DMiMD

**Figure 3:**
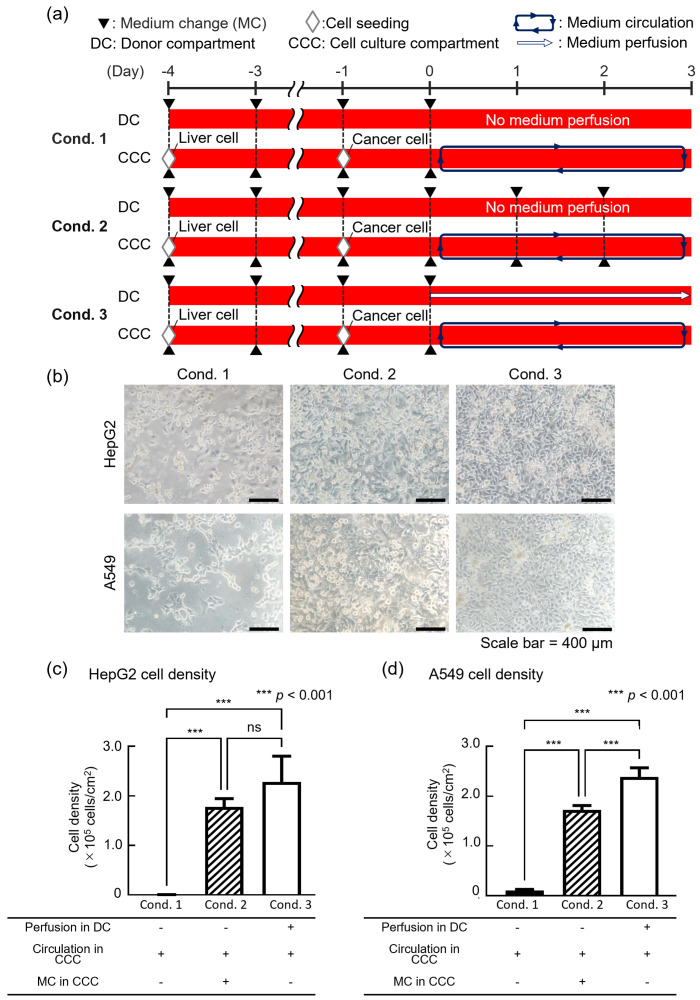
Evaluation of the nutrient supply performance of the DMiMD. (**a**) Experimental process for co-culture using the DMiMD. (**b**) Photographs of HepG2 and A549 cells after perfusion co-culture using the DMiMD. Cell morphology was observed using different medium change methods. (**c**,**d**): Cell density of HepG2 and A549 cells with different medium change methods when co-cultured using the DMiMD. The cell densities of both HepG2 (**c**) and A549 (**d**) indicated that the medium change method using a dialysis membrane (cond. 3) provided nutrient supply equivalent to or better than that provided using the conventional method (cond. 2). “ns” means no significant.

We evaluated cell viability with different medium change methods when co-culturing liver and lung cancer model cells using the DMiMD to investigate whether cell culture was possible with nutrient supply through the dialysis membrane. In this experiment, HepG2 as liver model cells and A549 as lung cancer model cells were co-cultured in each organ model chamber of the DMiMD. DMEM high glucose (12430-054, Dulbecco’s Modified Eagle Medium, Thermo Fisher Scientific-JP, Tokyo, Japan) was used as the medium. The following three experimental conditions were implemented: Condition 1 (cond. 1) involved no medium perfusion in the DC, without medium change in the CCC medium as the control. Condition 2 (cond. 2) involved no medium perfusion in the DC, with medium change every 24 h in the CCC. Condition 3 (cond. 3) involved medium perfusion in the DC without medium change in the CCC. HepG2 cells were pre-cultured for 4 days, and A549 cells were pre-cultured for 1 day. Thereafter, the medium in the CCC was perfused for 3 days using the stirrer-based micropump (Figure 3a).

In cond.1, only a few HepG2 and A549 cells were present on day 3 of co-culture at the endpoint. In cond. 2, HepG2 and A549 cells were confluent; however, they were slightly larger. In cond. 3, HepG2 and A549 cells were confluent and showed a pavement-like morphology (Figure 3b). The cell density of both HepG2 and A549 was lowest in cond. 1, which was the control (Figure 3c,d). No significant difference in the cell density of HepG2 was found between cond. 2 and cond. 3, although the cell density in cond. 3 was higher. The cell density of A549 was significantly higher in cond. 3 than in cond. 2.

The glucose consumption rate of HepG2 is 37 µg/(h·10^−6^ cells) [20]; in comparison, that of A549 is 27 µg/(h·10^−6^ cells) [21]. In this experiment, assuming that HepG2 and A549 cells would not proliferate from the seeding density, glucose depletion was expected after roughly 53 h of co-culture. In cond. 1, the extremely low cell density of both HepG2 and A549 suggested glucose depletion in the medium. In cond. 2, glucose was supplied above the predicted consumption levels, 270 µg/((HepG2 1.0 × 10^5^ cells + A549 5.0 × 10^4^ cells)·day) of HepG2 and A549, by changing the medium in the CCC every 24 h. However, in cond. 2, the cell density of HepG2 and A549 was lower compared to cond. 3. This finding suggested that glucose consumption by cells in the CCC increased with cell proliferation so that the glucose level in the CCC might not have been sufficient for cell culture. In cond. 3, fresh medium was pumped to the DC, supplying glucose to the CCC through the dialysis membrane at the NSP. This condition may have contributed to the healthy culture of HepG2 and A549 cells. In the DMiMD, fresh culture medium was continuously delivered to the DC via a syringe pump, resulting in a continuous supply of glucose. Meanwhile, a stirrer pump perfused the internal medium in the CCC. This configuration maintained glucose in the DC; in the CCC, the glucose concentration decreased due to cellular consumption. As a result, a stable concentration gradient was established across the dialysis membrane, promoting efficient diffusion of glucose into the CCC. Furthermore, the continuous flow of medium on both sides of the membrane in the DC and the CCC prevented stagnation at the membrane surfaces and enhanced glucose diffusion efficiency. Incidentally, because the pore size of the dialysis membrane was extremely small and the flow resistance was sufficiently low, the effect of substance diffusion due to the pressure difference between the DC and the CCC could be ignored. Based on these findings, fresh medium was pumped to the DC in the DMiMD, supplying sufficient glucose to the CCC to maintain the cells. The findings presented above demonstrate that nutrient supply equivalent to or better than that obtained using the conventional method of regularly changing the entire medium is possible.

### 3.3. Evaluation of Anticancer Drug Efficacy Using the DMiMD

**Figure 4:**
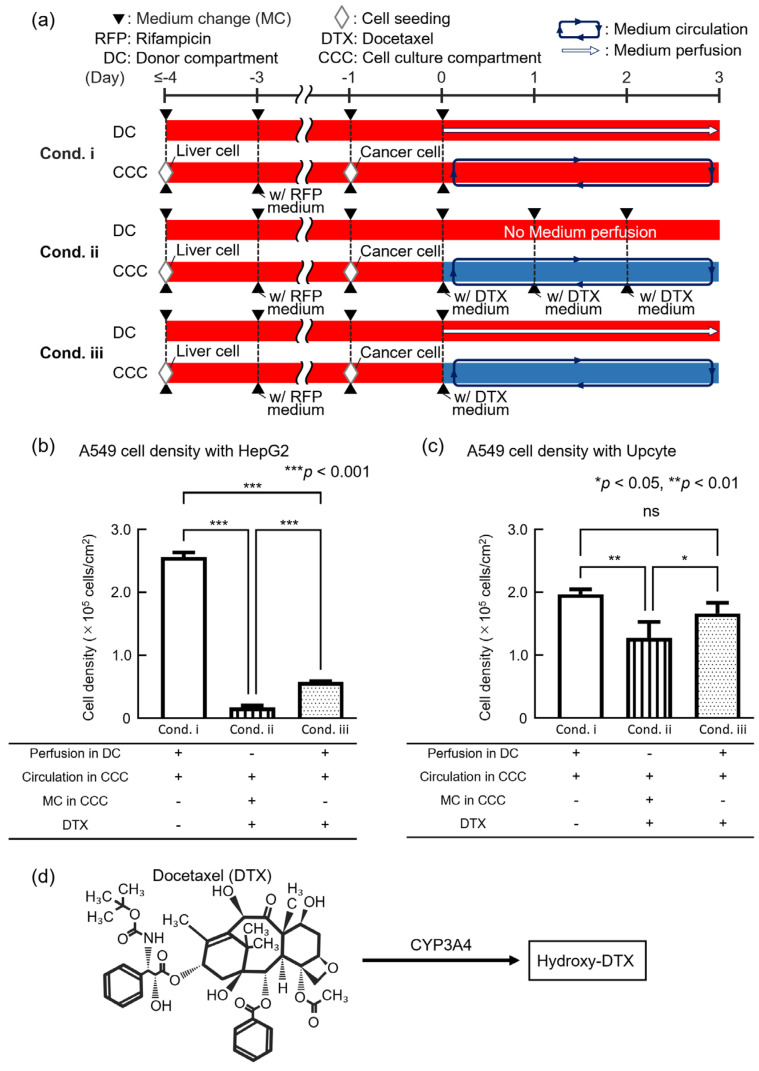
Evaluation of the efficacy of the anticancer drug Docetaxel (DTX) using the DMiMD. (**a**) An experimental process for an anticancer drug efficacy test using the DMiMD. (**b**,**c**) Cell density of A549 cells with different medium change methods when exposed to DTX with HepG2 (**b**) or Upcyte (**c**). (**d**) Chemical structure of DTX and major biotransformation pathways. The anticancer drug DTX is metabolized to hydroxylated derivatives by CYP3A4 in the liver and detoxified. “ns” means no significant.

We conducted a drug efficacy test with an anticancer drug to validate the utility of the DMiMD. In this experiment, liver model cells and anticancer drug-targeted cells were co-cultured in the DMiMD and exposed to the anticancer drug Docetaxel (DTX, 047-31281, FUJIFILM Wako, Osaka, Japan). HepG2 or Upcyte cells were used as liver model cells, and A549 cells were used as anticancer drug-targeted cells. When co-culturing Upcyte and A549, the DMiMD with a tissue-culture-treated dish plate was used due to the weak adhesion of Upcyte. The following three experimental conditions were implemented: Condition i (cond. i) involved medium perfusion in the DC without DTX as the negative control. Condition ii (cond. ii) involved DTX medium change in the CCC. Condition iii (cond. iii) involved fresh medium perfusion in the DC with no DTX medium change in the CCC. The exposure time for 0.1 μM DTX was 3 days (Figure 4a).

In the case of HepG2, the cell density of A549 in cond. ii_HepG2 and cond. iii_HepG2 was significantly lower than that in cond. i_HepG2 (negative control). A549 cell density in cond. iii_HepG2 was significantly higher than in cond. ii_HepG2 (Figure 4b). In the case of Upcyte, the cell density of A549 in cond. ii_Upcyte was significantly lower than that in cond. i_Upcyte. However, the cell density of A549 in cond. iii_Upcyte showed no significant difference compared to cond. i_Upcyte (negative control). The cell density of A549 in cond. iii_Upcyte was significantly higher than that in cond. ii_Upcyte (Figure 4c). The trend of the cell density of A549 in cond. iii being higher than in cond. ii was consistent for both HepG2 and Upcyte. However, there was a clear difference in the cell density of A549 between cond. ii and cond. iii, with lower cell density in the case of HepG2 and higher cell density in the case of Upcyte.

DTX is a cytotoxic drug which exhibits potent anti-mitotic activity against a wide range of cancers, including lung cancer [22], DTX also promotes the polymerization of tubulin in cancer cells and inhibits the depolymerization of microtubules. Tubulin polymerized by DTX forms stable microtubules; however, these microtubules are prevented from depolymerizing, leading to the formation of abnormal microtubule bundles inside the cell. The abnormal formation of microtubules inside the cell inhibits normal division and halts cell mitosis. As a result, apoptosis is triggered [23,24]. DTX is metabolized by hepatic CYP3A4 into hydroxylated derivatives, resulting in detoxification (Figure 4d) [25,26]. Furthermore, DTX is known to bind to albumin, a high-molecular-weight protein in the blood [27]. DTX bound to albumin was predicted not to permeate through the dialysis membrane as its molecular weight exceeds the MWCO of the membrane. Therefore, in cond. iii, even though glucose was supplied from the DC to the CCC, DTX was expected to remain in the CCC. When HepG2 was employed, the lower cell density of A549 in cond. ii_HepG2 and cond. iii_HepG2 compared to the control cond. i_HepG2 suggested that DTX remained in the CCC and exhibited efficacy against A549.

When Upcyte was employed, the cell density of A549 in both cond. ii and cond. iii was higher than when HepG2 was used (Figure 4b,c). This finding could possibly be linked to the fact that Upcyte showed 45- to 143-times-higher CYP3A4 activity than HepG2, leading to enhanced detoxification of DTX [28]. In this experiment, although HepG2 was exposed to Rifampicin to induce CYP3A4, the original HepG2 cells exhibited low CYP3A4 expression; therefore, even after exposure to Rifampicin, they might not have produced sufficient CYP3A4 to detoxify DTX. Moreover, the cell density of A549 in cond. iii_Upcyte was significantly higher than in cond. ii_Upcyte and was equivalent to that in the negative control (cond. i_Upcyte), which was not exposed to DTX. This finding is likely due to the fact that the area under the concentration–time curve (AUC) of DTX differed between cond. ii and cond. iii. The AUC of DTX for a period of 3 days differed between cond. ii, in which the medium in the CCC was refreshed every 24 h, and cond. iii, in which nutrients were supplied through a dialysis membrane. That is, the AUC in cond. ii was considered to be larger than that in cond. iii. As a result, it was considered that sufficient drug efficacy was not observed in cond. iii_Upcyte because Upcyte metabolized DTX. This finding can be linked to the reproduction of continuous drug concentration changes similar to those in vivo. In conventional cell culture methods, drug concentrations are reset with each medium change, making it difficult to reproduce in vivo conditions and possibly leading to inaccurate drug efficacy evaluation.

Based on these findings, the DMiMD was shown to retain drugs in the CCC when the medium change method was applied through dialysis membranes, enabling the evaluation of anticancer drug efficacy under continuously changing drug concentration conditions. The retention performance of the dialysis membrane largely depends on the drug’s molecular weight or its protein-bound complex. DTX strongly binds to albumin in the culture medium and, as a result, forms a high-molecular-weight complex. This was effectively retained by the membrane with an MWCO of 3.5 × 10^3^. Similarly, drugs with high protein-binding affinity are also retained, whereas drugs with low molecular weight and low protein-binding affinity pass through the dialysis membrane. Furthermore, the dialysis membrane used in the DMiMD enables the integration of membranes with different MWCOs, providing flexibility to accommodate drugs with various molecular sizes and protein-binding properties.

Although the observed decrease in cancer cell density supports the retention of DTX within the DMiMD, the current evaluation method has several limitations. If the concentration of DTX could be directly quantified, it would provide stronger evidence for verifying the retention performance of the dialysis membrane. However, because the microfluidic channel and chamber used in this study have a closed-system structure, it was technically difficult to sample the drug concentration during the experiment. Therefore, we used biological responses, such as decrease in cancer cell density, as an indirect indicator of drug retention. In addition, quantification through the use of LC-MS/MS was also difficult under these conditions. In the future, it will be necessary to introduce labeled detection methods, such as fluorescent labeling, to enable more direct evaluation of the retention mechanism. Although we did not include a direct comparison with in vivo pharmacokinetic profiles in this study, such comparative analysis would be beneficial for future validation of the physiological relevance of the DMiMD. In addition, although we did not conduct a systematic evaluation of device stability and repeatability, the experimental results were consistent across multiple replicates under the same experimental conditions. These results indicate that the DMiMD exhibits stable performance. We concluded that the DMiMD can serve as a useful tool for drug efficacy testing.

## 4. Conclusions

In this study, we developed the DMiMD as a novel in vitro drug efficacy test system to achieve drug concentration changes similar to those obtained under in vivo conditions. The DMiMD integrates a dialysis membrane between the CCC and the DC, facilitating exposure to an anticancer drug under concentration changes similar to in vivo conditions through nutrient supply via the dialysis membrane. The molecular selectivity of the DMiMD was evaluated using substances of different molecular weights. The results demonstrated that the DMiMD functions via selective permeation and retention of substances based on molecular weight. Comparing nutrient supply methods in the co-culture of HepG2 and A549 cells illustrated that introducing fresh medium to the DC can supply sufficient glucose to the cultured cells through the dialysis membrane. The results for co-culturing cells for metabolism and for cells as the target of drugs and exposure to the anticancer drug DTX demonstrate that the DMiMD functions as an in vitro drug efficacy testing system. From our experimental findings, we were able to confirm that the nutrient supply methods through a dialysis membrane can be used to evaluate the efficacy of anticancer drugs under continuously changing drug concentrations. These results indicate that the DMiMD functions as an in vitro drug efficacy testing system that provides continuous glucose supply to cultured cells and achieves drug concentration changes similar to in vivo conditions. The DMiMD, which integrates the nutrient supply methods for retaining anticancer drugs, is expected to be an innovative tool in the fields of drug discovery and biology because of its function as an in vitro drug efficacy testing system which delivers performance similar to in vivo conditions.

## Data Availability

The original contributions presented in this study are included in the article. Further inquiries can be directed to the corresponding author.
